# Chrysin targets myeloid‐derived suppressor cells and enhances tumour response to anti‐PD‐1 immunotherapy

**DOI:** 10.1002/ctm2.1019

**Published:** 2022-09-19

**Authors:** Yinan Li, Ru Yang, Xiu Huang, Caihong Chen, Daolei Dou, Qianqian Wang, Xinying Wu, Huijuan Liu, Tao Sun

**Affiliations:** ^1^ State Key Laboratory of Medicinal Chemical Biology and College of Pharmacy Nankai University Tianjin China

1

Dear Editor,

Myeloid‐derived suppressor cells (MDSCs) accumulate in large numbers during tumour development and help tumours evade the immune system.^1^, ^2^, ^3^ Cancer immunotherapy, such as immune checkpoint inhibitors, is the most promising therapeutic strategy currently.^4^ However, its efficacy varies significantly from individual to individual. The accumulation of MDSCs is a crucial factor in the low anticancer efficacy of PD‐1 inhibitors.^5^, ^6^ Some flavonoids can act on immune checkpoints.^7^, ^8^ Chrysin (Chr) is a natural and biologically active flavonoid with antioxidant, anti‐inflammatory and anticancer effects.^9^ Besides, Chr has a wide range of sources and high safety. It is a drug with great potential in cancer treatment. However, studies on Chr's regulation of the tumour microenvironment are still lacking. In this study, we reported the effect of Chr on tumour microenvironment, including immune infiltration and angiogenesis, and further explored the feasibility of combining chrysin and PD‐1 inhibitor.

Based on the IC_50_ of the Chr inhibitory effect in MDSCs was 43.79 µM (Figure 1A). We chose two representative doses for further study: 10 µM (low dose) and 20 µM (high dose). MDSCs were typically divided into two subgroups: granulocytic cells (G‐MDSC) and monocytic cells (M‐MDSC). The inhibitory effect of Chr on MDSCs mainly affected G‐MDSCs (Figure 1B). Apoptosis and proliferation were crucial for cell accumulation. Chr induced apoptosis and G0/G1 cell cycle arrest of MDSCs (Figure 1C,D). The detection of CFSE showed that Chr also inhibited the proliferation of MDSCs (Figure 1E). Arginase 1 (Arg‐1) and inducible nitric oxide synthase (iNOS) activities, as well as reactive oxygen species (ROS), are critical to the tumour immunosuppressive effect of MDSCs. Chr reduced the expressions of Arg‐1 and MDSCs proliferation‐inducing factor COX‐2 at the mRNA and protein levels and iNOS mRNA in a dose‐dependent manner (Figure 1F,G). The levels of NO and ROS produced by MDSCs and Arg‐1 activity were also significantly reduced after Chr treatment (Figure 1H–J). The previous results indicated that Chr mainly inhibited the accumulation of G‐MDSCs and their immunosuppressive activity.

**FIGURE 1:**
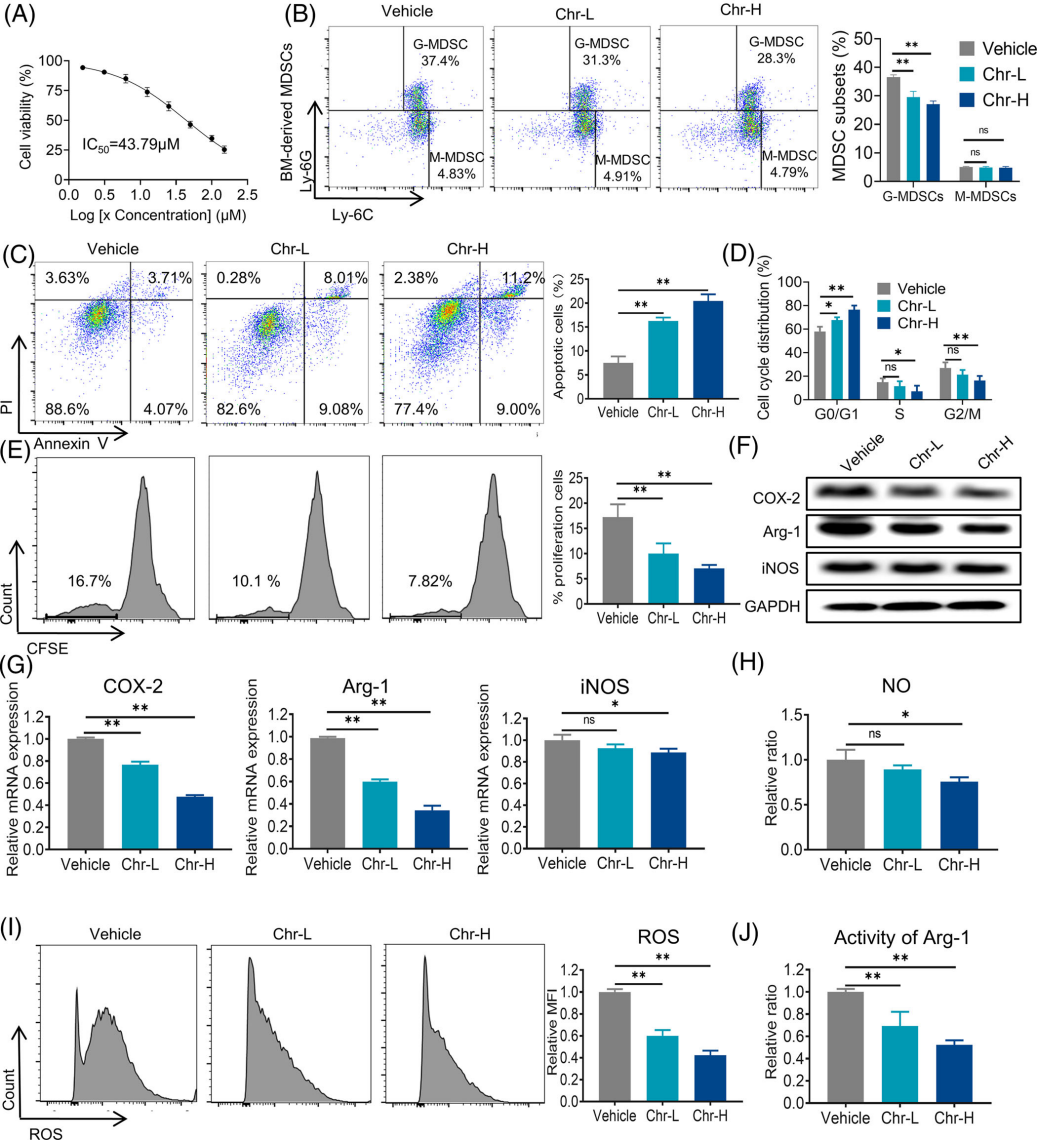
Chrysin (Chr) inhibits the accumulation and function of myeloid‐derived suppressor cells (MDSCs) in vitro: (A) CCK8 assay results of Chr on MDSCs; (B) effect of Chr on B16‐F10‐bearing C57BL6 mouse myeloid cells, as detected by flow cytometric analysis; (C) analysis of MDSCs apoptosis induced by Chr using Annexin V/PI assay; (D) effect of Chr on the cell cycle of MDSCs; (E) proliferation of CFSE‐labelled MDSCs, as determined by flow cytometry. Inducible nitric oxide synthase (iNOS), COX‐2, and Arg‐1 protein (F) and mRNA (G) levels in Chr‐treated MDSCs. Immunosuppressive function analysis of MDSCs after Chr treatment, including NO production (H), reactive oxygen species (ROS) level (I) and Arg‐1 activity (J). Data expressed as mean ± SD, *n* = 3. ns, not significant. *p < .05, **p < .01. Chr‐L, 10 µM; Chr‐H, 20 µM

To determine the effects of Chr on tumour progression, melanoma (B16‐F10) was established in C57BL/6 mice (Figure 2A). Chr treatment significantly decreased the tumour volume (Figure 2B,D) and tumour weight (Figure 2E) but did not affect the body weight (Figure 2C). Flow cytometric analysis showed that Chr inhibited the accumulation of G‐MDSCs in the marrow and spleen of tumour‐bearing mice (Figures 2F and S1A,C). MDSCs inhibited the proliferation and functional activity of T cells. Further analysis of CD8^+^ T cells showed that high‐dose Chr treatment could alleviate the inhibition of T cell proliferation caused by MDSCs (Figures 2G and S1B,D). The previous results demonstrated that Chr can inhibit the accumulation of G‐MDSCs and restore T cell proliferation, thereby inhibiting the malignant progression of tumours.

**FIGURE 2:**
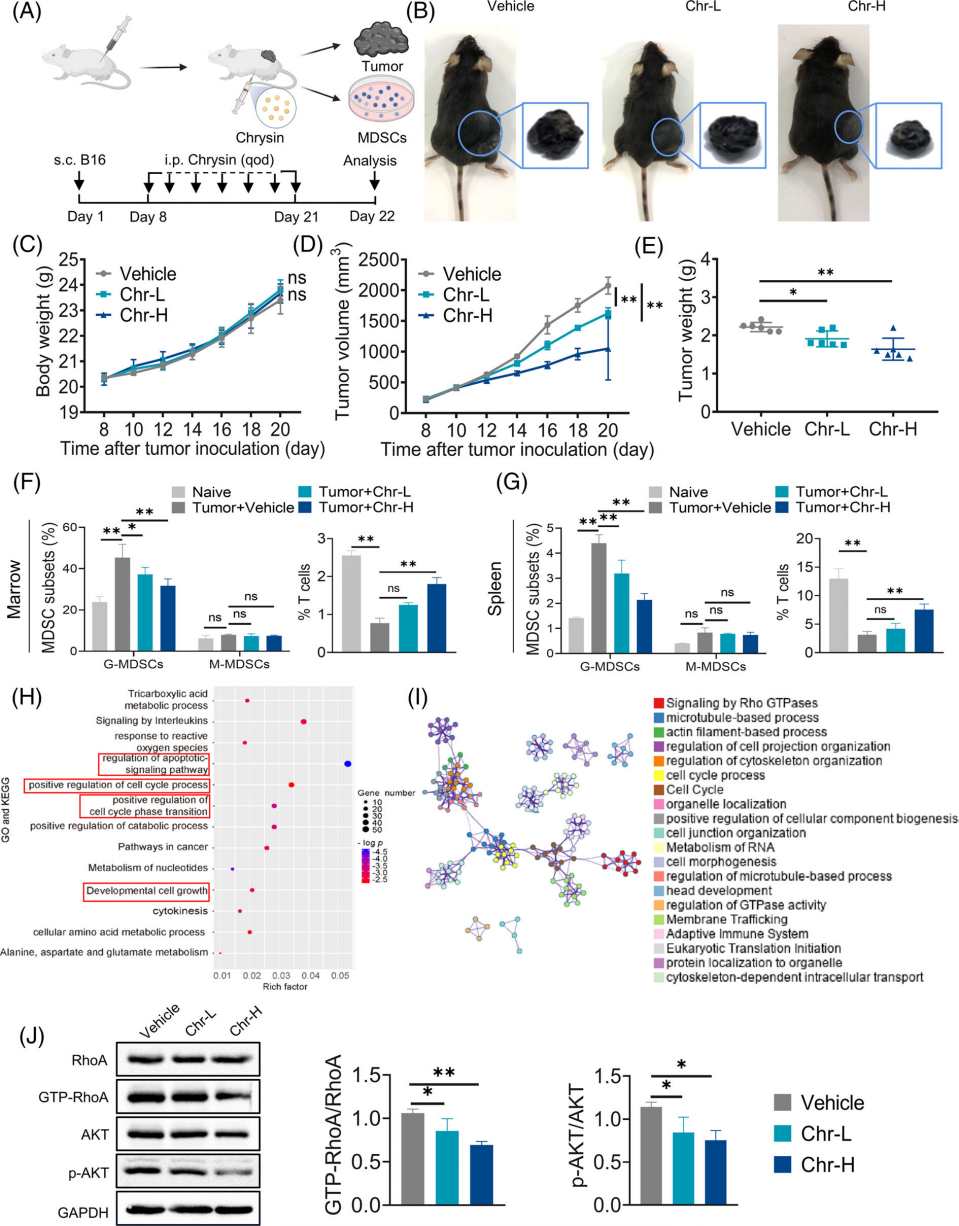
Chrysin targeting RhoA/PI3K/AKT pathway inhibits myeloid‐derived suppressor cells (MDSCs) and exerts anti‐tumour effect: (A) Schematics show the treatments of tumour‐bearing C57BL6 mice; (B) representative photographs of transplanted tumours at the end of the experiment; mouse body weight (C), tumour volume (D) and tumour weight (E) change in each group; flow cytometry analysed the effect of Chr on the ratio of MDSCs (left) and CD8^+^ T cells (right) in the marrow (F) and spleen (G). Chr‐L, 20 mg/kg; Chr‐H, 40 mg/kg, *n* = 6; (H) enrichment analysis of GO and KEGG; (I) protein–protein interaction (PPI) network of differential genes in MDSCs; (J) the effects of Chr on RhoA, Akt and p‐Akt in G‐MDSC at the protein level, as detected by Western blot. Data expressed as mean ± SD. ns, not significant. *p < .05, **p < .01. Chr‐L, 10 µM; Chr‐H, 20 µM

To further understand the mechanism underlying Chr's regulation of MDSCs, proteomic analyses were performed. GO and KEGG pathway enrichment analysis supported the effects of Chr on various pathways, such as cell apoptosis, cell cycle and cell proliferation (Figure 2H). Protein–protein interaction enrichment suggested that Chr may influence the accumulation and function of MDSCs by affecting signalling by Rho GTPases (Figure 2I). Ras homolog gene family member A (RhoA) is a staple small GTPase protein in the Rho family. RhoA can regulate the classical pathway PI3K/AKT that is closely related to cell proliferation and apoptosis. Western blot showed that Chr inhibited the activation level of RhoA and the phosphorylation of AKT (Figure 2J). Furthermore, Akt activators restored Chr's inhibitory effects on ROS levels and Arg‐1 activity, and Akt inhibitors had no significant effect on Chr's ROS levels and Arg‐1 activity inhibition (Figure S2). The previous results supported that Chr inhibits the accumulation and immunosuppressive activity of MDSCs by targeting the RhoA/PI3K/AKT pathway.

MDSCs exert immunosuppression and also affect tumour angiogenesis. Therefore, we focused on the angiogenesis‐related part of the proteomic analysis. The heat maps showed that Chr inhibited the promotion of tumour angiogenesis by MDSCs (Figure 3A). Flow cytometry analysis indicated that Chr treatment decreased G‐MDSCs in blood and tumours, whereas CD8^+^ T cell tumour infiltration increased (Figure 3B–D). Vascular abnormalities always lead to tumour hypoxia, and hypoxia‐inducible factor (HIF)‐1α is a major gene involved in coordinating tumour cell adaptation to hypoxia and promoting angiogenesis. Immunohistochemistry (IHC) showed that Chr reduced HIF‐1α expression levels in tumours (Figure 3E). CD31 immunostaining allowed the visualization of tumour vessels; when combined with dextran and lectin, it helped assess tumour vessel permeability and perfusion levels, respectively. The results showed that microvessel density decreased, vascular permeability decreased and vascular perfusion increased in tumour after Chr administration (Figure 3F). Treatment with Chr also dose dependently induced the apoptosis of the tumour cells (Figure 3G). Haematoxylin and eosin staining indicated increased intratumoural immune infiltration and enlarged tumour necrosis area after Chr administration (Figure 3H).

**FIGURE 3:**
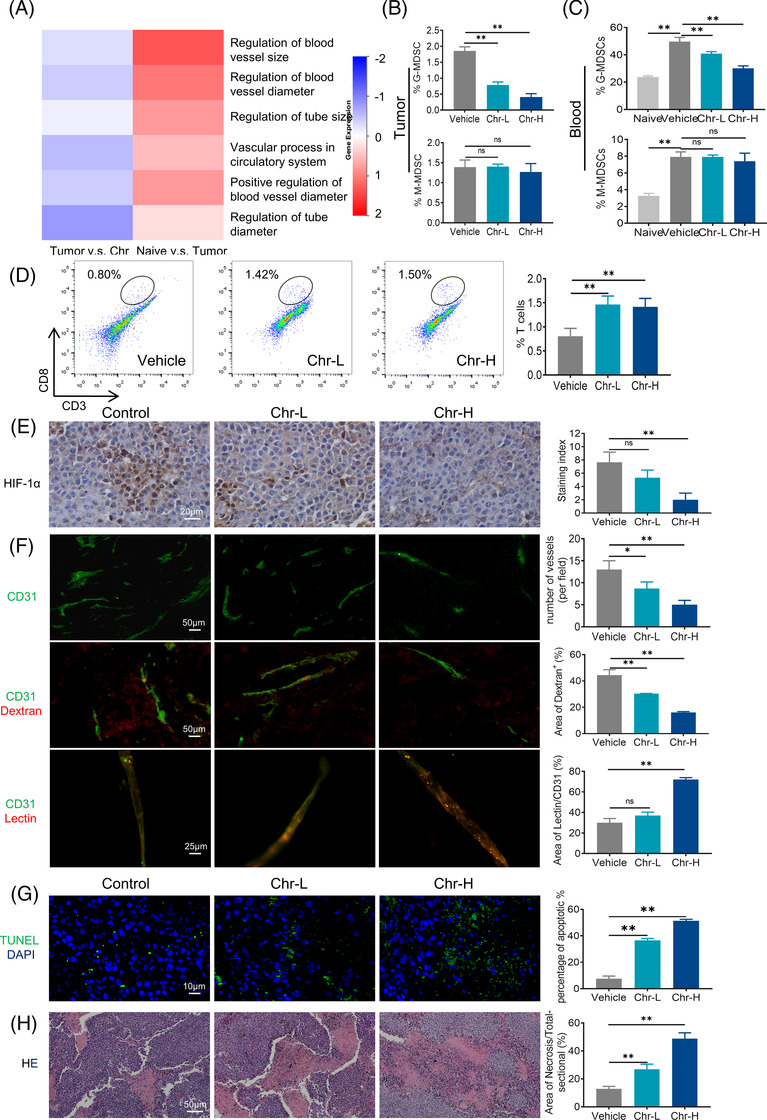
Chrysin inhibits the promotion capability of myeloid‐derived suppressor cells (MDSCs) on tumour angiogenesis in vivo: (A) proteomic analysis of angiogenesis‐related pathways; (B and C) flow cytometric analysed the ratio of MDSCs in tumour and peripheral blood of B16‐F10 tumour‐bearing mice after Chr treatment; (D) flow cytometry was used to analyse CD8^+^ T cells infiltration in tumour tissues after Chr treatment; (E) representative image of immunohistochemistry (IHC) analysis of hypoxia‐inducible factor (HIF)‐1α expression in tumour tissues; (F) microvascular densities, vascular permeability and vascular perfusion changes in tumours of each group; (G) apoptosis evaluation by TUNEL assay in each group; (H) representative haematoxylin and eosin (HE) staining of tumours sections in each group. Quantification was performed on three random visual fields for each sample (three sample per group). Data expressed as mean ± SD. ns, not significant. *p < .05, **p < .01. Chr‐L, 20 mg/kg; Chr‐H, 40 mg/kg

The PD‐1/PD‐L1 axis inhibited the anti‐tumour immunity of T cells. We tried to combine Chr with PD‐1 inhibitor to suppress MDSC‐mediated inhibition of T cell proliferation while supporting sufficient T cell activity. Flow cytometry analysis confirmed that treatment with Chr and PD‐1 inhibitor decreased MDSCs and increased T cells compared with the single drug group (Figure S3). In vivo, after the combination treatment, the tumour volume and tumour weight were smaller, the mice survived better in the long term and the mice's body weight remained stable (Figure 4A–E). Further, the triple‐negative breast cancer cells (4T1)–BALB/C mice model was implemented to determine whether Chr could broadly enhance the effects of PD‐1 inhibitor. Continuous monitoring showed that combination therapy with Chr and PD‐1 inhibitor outperformed monotherapy in inhibiting tumour growth (Figure 4F,H,I) and prolonging survival (Figure 4J) without affecting body weight (Figure 4G). IHC revealed a decrease in the expression levels of Ki67 and PD‐L1 after the drug combination (Figure S4).

**FIGURE 4:**
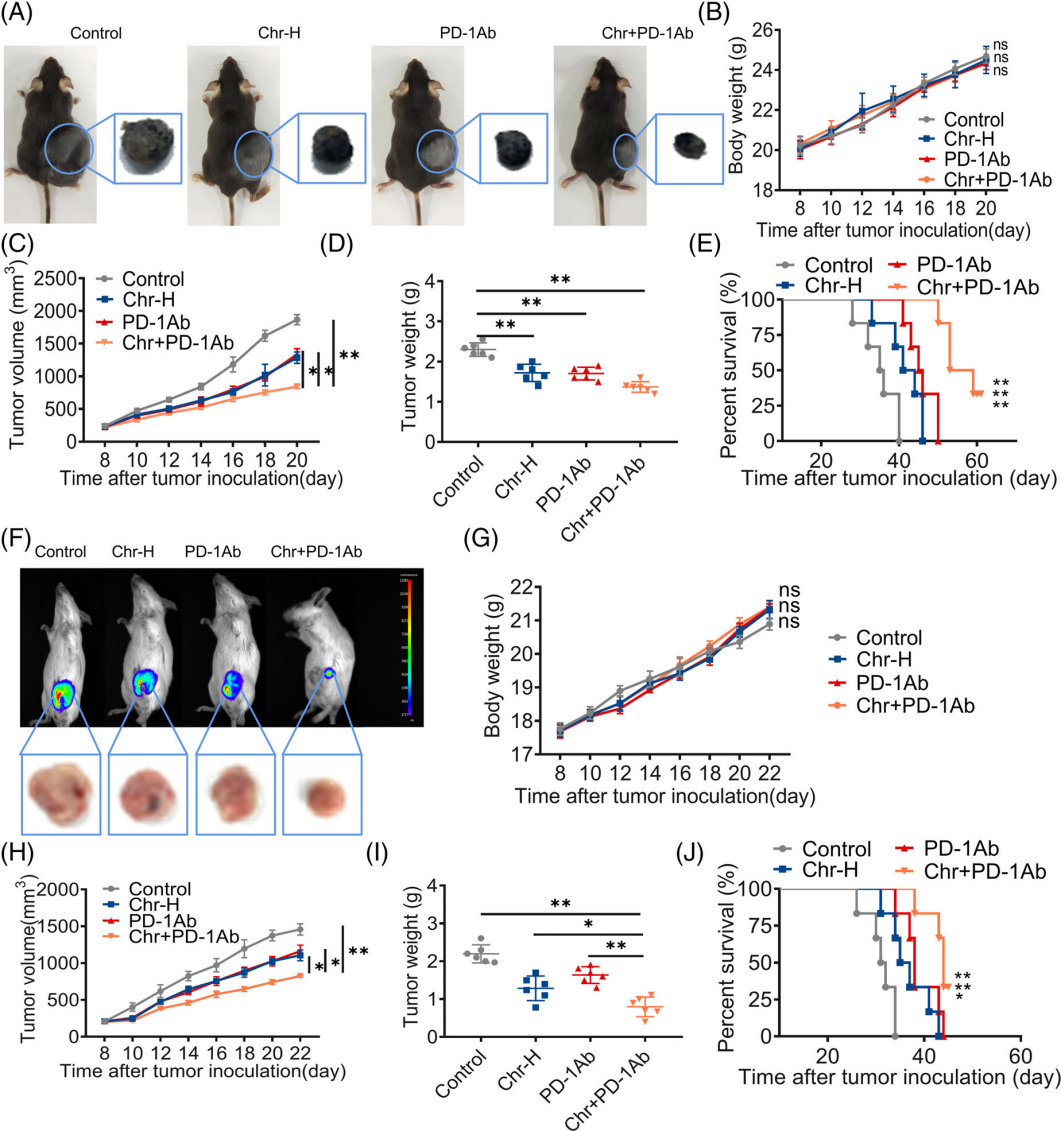
Chrysin synergistically enhances the anti‐tumour activity of PD‐1 inhibitors: (A) representative photographs of transplanted tumours at the end of the experiment; change in mouse body weight (B), tumour volume (C) and tumour weight (D) after administration of Chr and PD‐1 Ab, whether alone or in combination; (E) survival curve of B16‐F10 tumour‐bearing mice after Chr and PD‐1 Ab were administered individually or in combination; (F) analysis of 4T1‐Luc tumour by live‐animal fluorescence imaging; change in mouse body weight (G), tumour volume (H) and tumour weight (I) after the administration of Chr and PD‐1 Ab in 4T1 tumour‐bearing BALB/C mice; (J) survival curve of 4T1 tumour‐bearing BALB/C mice after Chr and PD‐1 mAb were administered individually or in combination. Data expressed as mean ± SD, *n* = 6. ns, not significant. *p < .05, **p < .01. Chr‐H, 40 mg/kg; PD‐1 Ab, 10 mg/kg; Chr, 40 mg/kg + PD‐1 Ab, 10 mg/kg

In conclusion, Chr can inhibit the accumulation and function of MDSCs by targeting RhoA/PI3K/AKT pathway, and the combination of chrysin and PD‐1 inhibitor can be an anticancer strategy (Figure S5).

## CONFLICT OF INTEREST

The authors declare that they have no competing interests.

## FUNDING INFORMATION

This work was supported by the National Natural Science Funds of China (Grant nos. 82073205, 81872374 and 81871972), the Fundamental Research Funds for the Central Universities, Nankai University, Hundred Young Academic Leaders Program of Nankai University, the Natural Science Foundation of Tianjin (19JCJQJC63200 and 21JCZDJC00930), the National Youth Talent Support Program (2020), and China Postdoctoral Science Foundation (No. 2021M701778).

## Supporting information

Supporting Information
