# Supplementary material for: Chrysin targets myeloid‐derived suppressor cells and enhances tumour response to anti‐PD‐1 immunotherapy
Source: Clin Transl Med. 2022 Sep 19;12(9):e1019. doi: 10.1002/ctm2.1019 (PMC9484264; doi:10.1002/ctm2.1019)
Supplement: Supplementary file 1 — Supporting Information [file CTM2-12-e1019-s001.docx]

Supporting Information

**Chrysin targets myeloid-derived suppressor cells and enhances tumor response to anti-PD-1 immunotherapy**

Yinan Li^1,#^, Ru Yang^1,#^, Xiu Huang^1^, Caihong Chen^1^, Daolei Dou^1^, Qianqian Wang^1^, Xinying Wu^1^, Huijuan Liu^1,^*, Tao Sun^1,^*

^1^State Key Laboratory of Medicinal Chemical Biology and College of Pharmacy, Nankai University, Tianjin, China.

^#^These authors contributed equally to this work as co-first authors.

*Corresponding author Email: tao.sun@nankai.edu.cn (T.S.); huijuan.liu@nankai.edu.cn (H.L.)

**
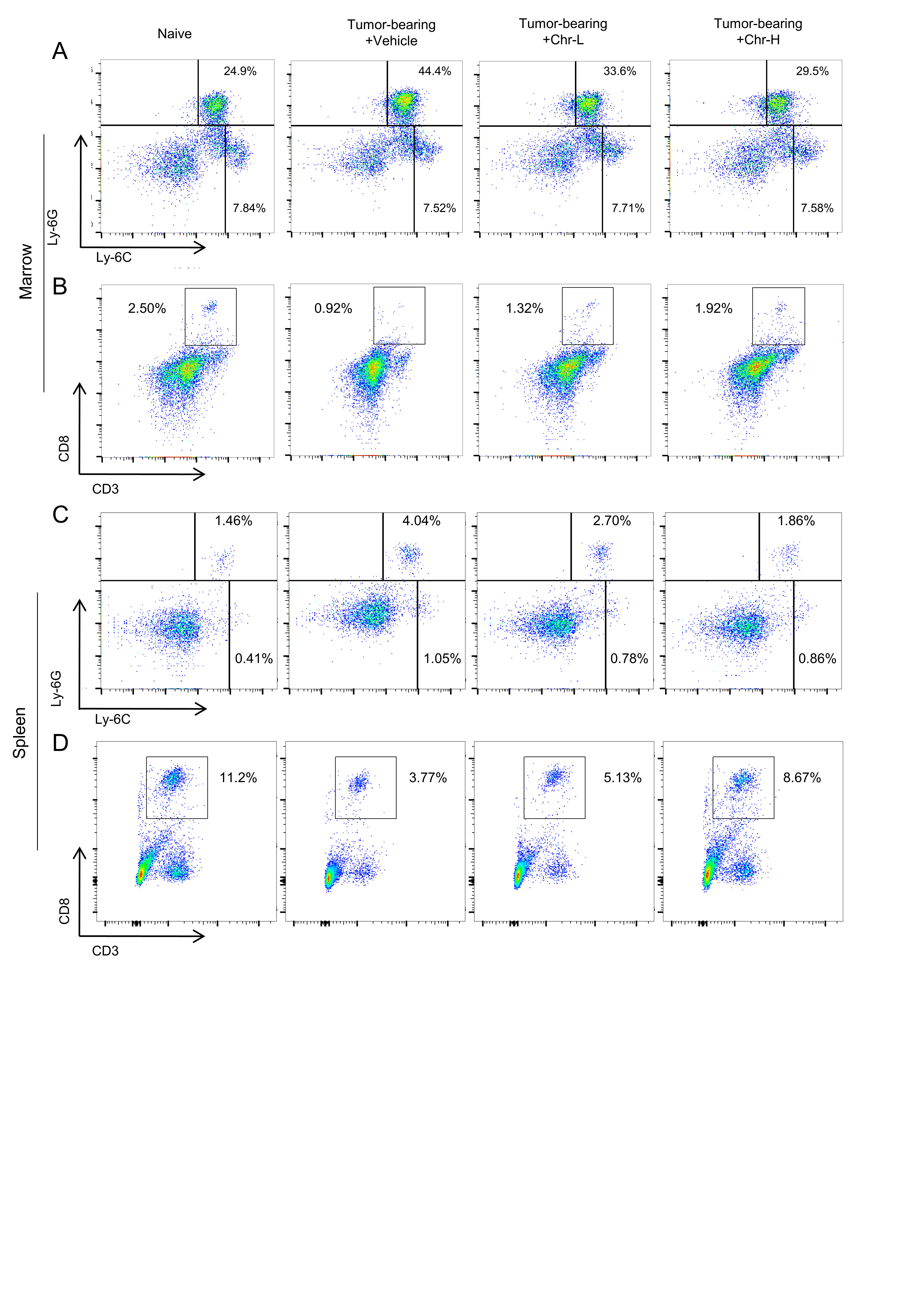
**

**Figure S1.** A–B) Representative flow cytometry plots of MDSCs (A) and CD8^+^ T cells (B) in the marrow of B16-F10 tumor-bearing C57BL6 mice after Chr treatment. C-D) Representative flow cytometry plots of MDSCs (C) and CD8^+^ T cells (D) in the spleen after Chr treatment.


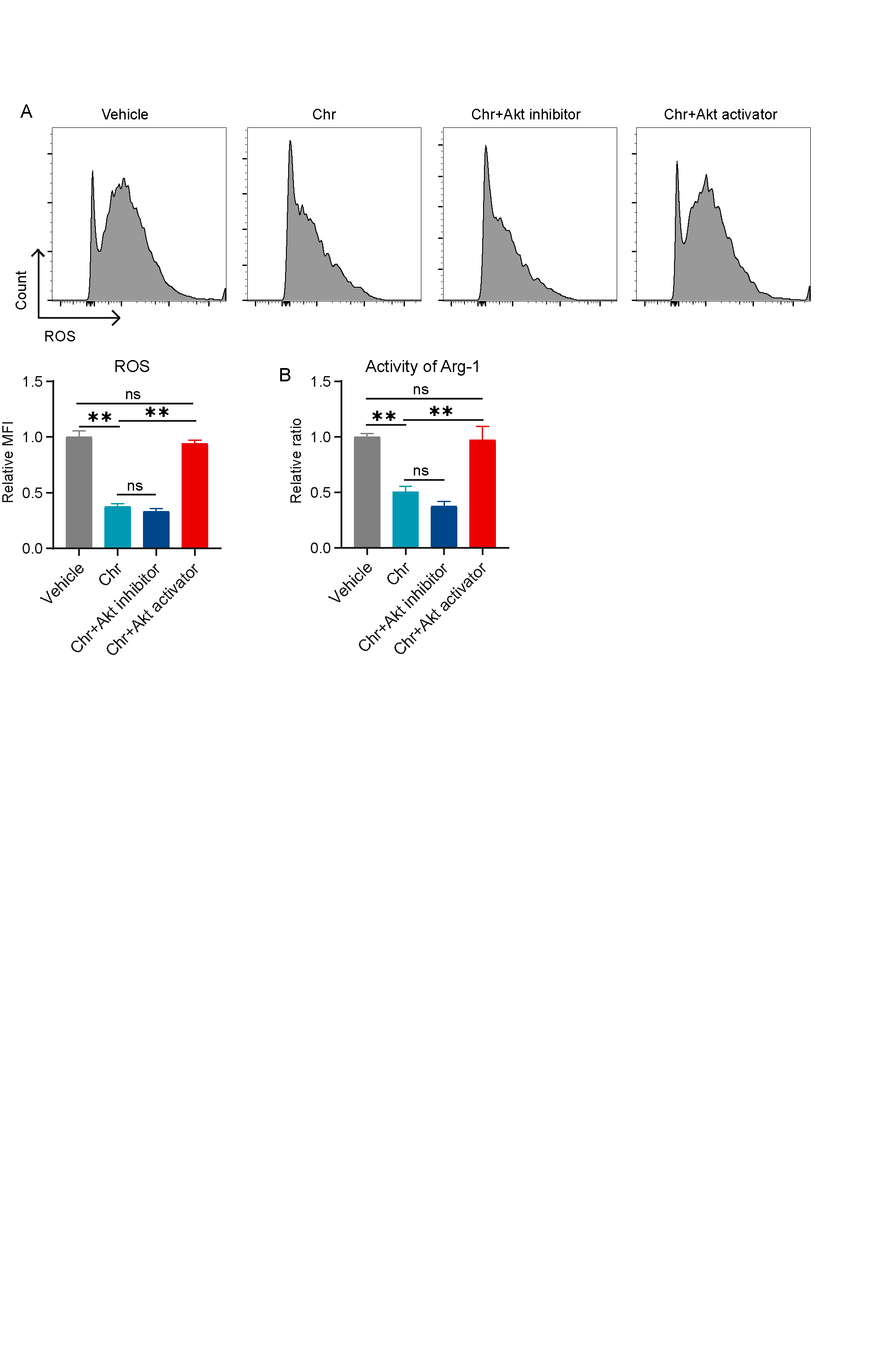


**Figure S2.** A) Flow cytometry analysis of ROS levels in MDSCs under the treatment of Vehicle, Chr, Chr+Akt inhibitor, and Chr+Akt activator. B) Arginase activity measured in MDSCs under the treatment of Vehicle, Chr, Chr+Akt inhibitor, and Chr+Akt activator.

**
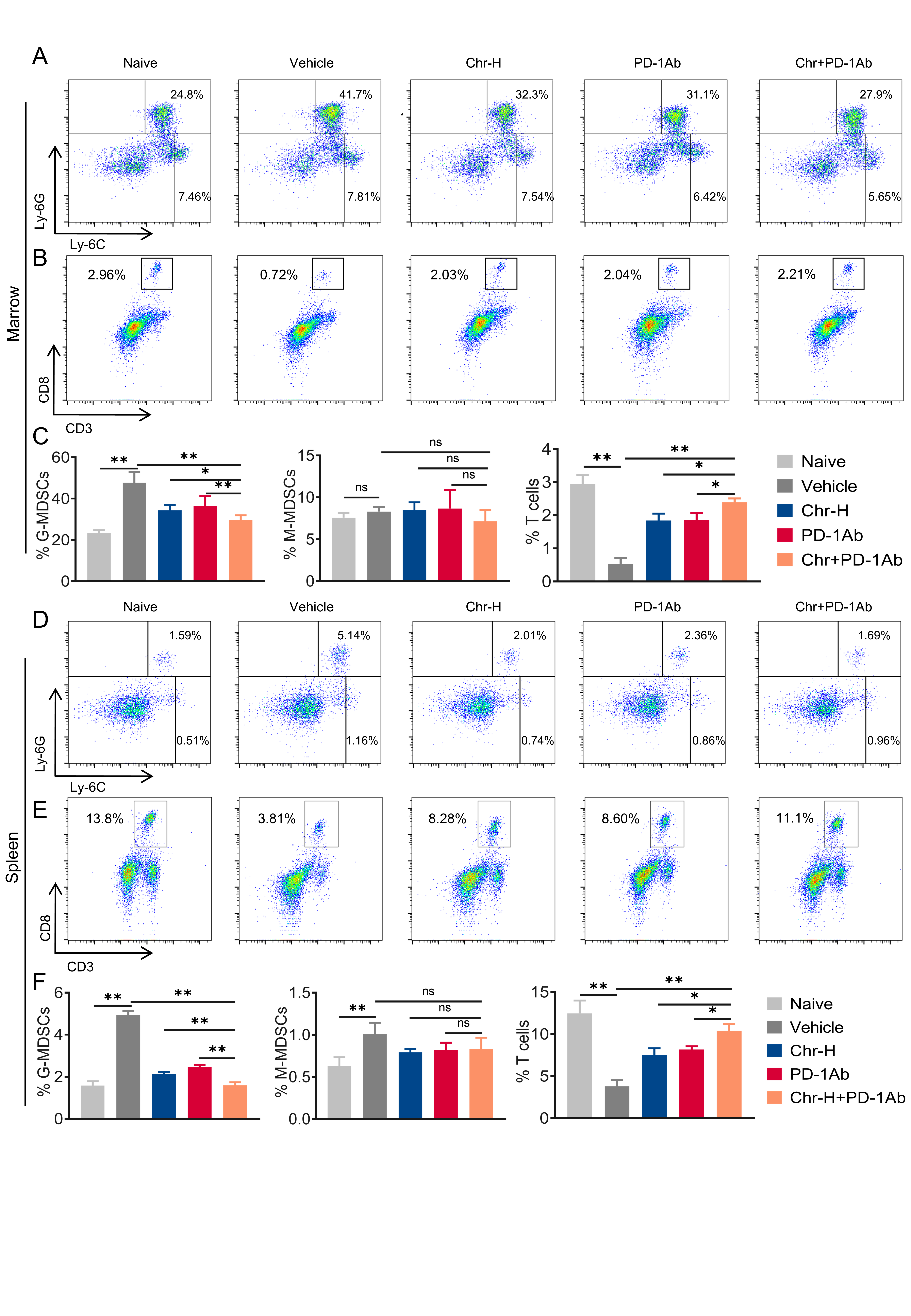
**

**Figure S3.** A–C) Flow cytometry was used to analyze the effect of Chr and PD-1 inhibitor on the ratio of MDSCs (A) and CD8^+^ T cells (B) in the marrow of B16-F10 tumor-bearing C57BL6 mice and statistical analysis (C). D–F) Flow cytometry was used to analyze the effect of Chr and PD-1 inhibitor combination on the ratio of MDSCs (D) and CD8+ T cells (E) in the spleen of B16-F10 tumor-bearing C57BL6 mice and statistical analysis (F). Data expressed as mean ± SD, n.s., not significant, *P < 0.05, **P < 0.01.

**
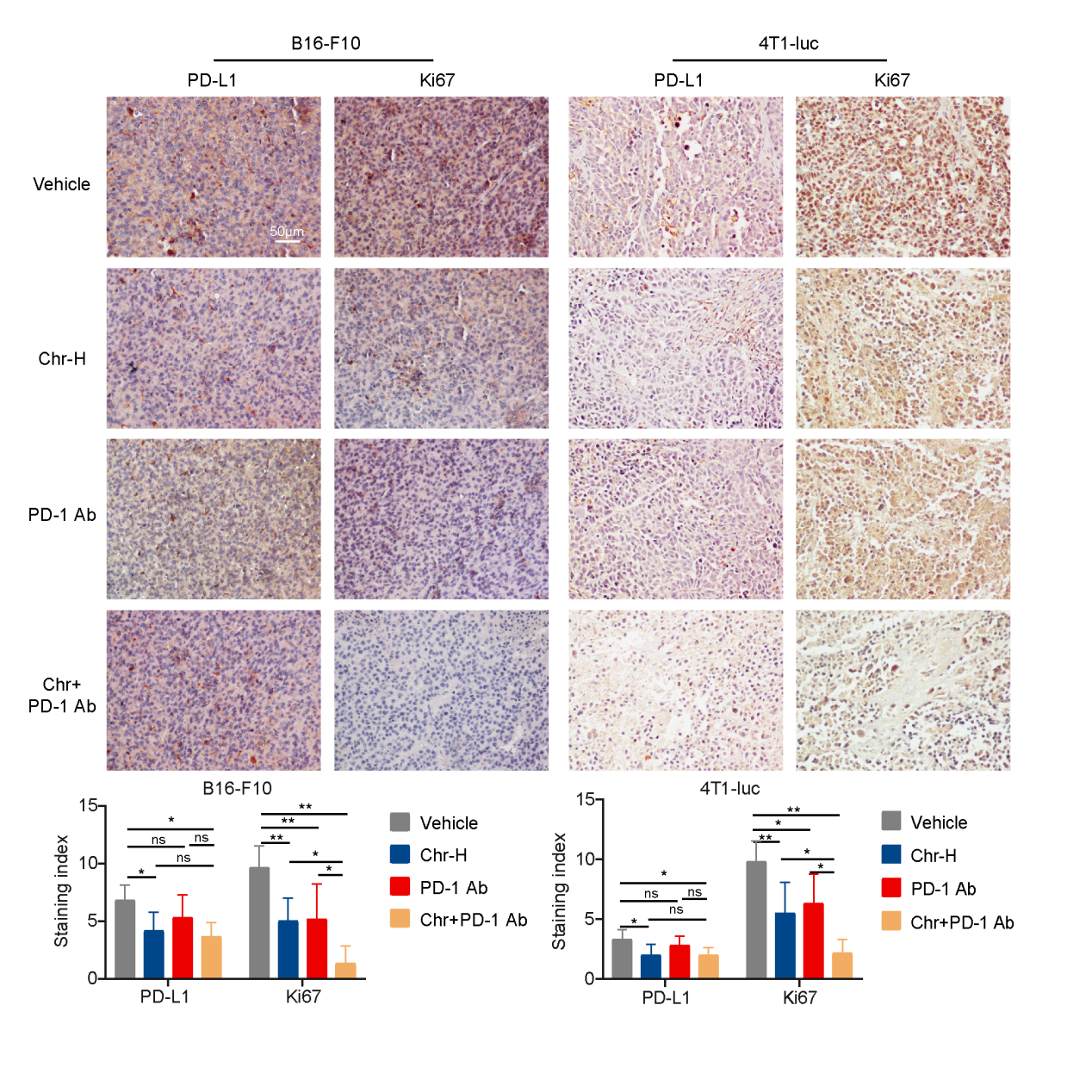
**

**Figure S4.** Immunohistochemical staining of Ki67 and PD-L1. Quantification was performed on three random visual fields for each sample (three sample per group). Data expressed as mean ± SD, n.s., not significant, *P < 0.05, **P < 0.01.


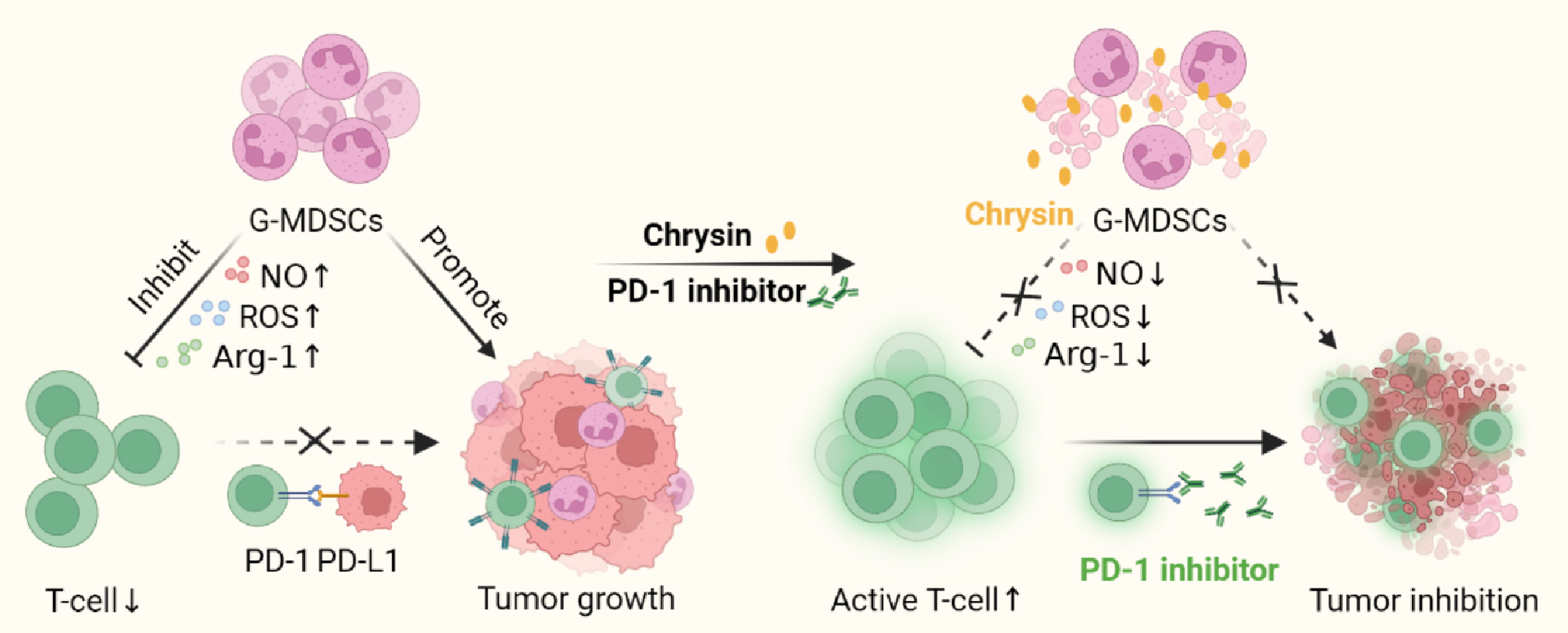


**Figure S5.** Schematic of chrysin targeting MDSCs to enhance tumor response to anti-PD-1 immunotherapy.

**Materials and methods**

**Cell culture**

B16-F10 and 4T1 (KeyGen Biotech, China) were further cultured in 1640 medium (Gibco) supplemented with 10% fetal bovine serum (Hyclone) in a 37 °C incubator with 5% CO_2_.

**Animals**

Female C57BL/6 and BALB/C mice (6 weeks old) were purchased from Charles River and housed under standard conditions. All animal experiments conformed to the guidelines of the Animal Ethics Committee of the Tianjin International Joint Academy of Biotechnology and Medicine.

**Mouse subcutaneous tumor-bearing model**

Subcutaneous B16-F10 or 4T1 tumor model was established by injecting the cells (4 × 10^5^) in a volume of 100 μL PBS into the right lateral flank of the mice. When the tumor volume reached ~150  mm^3^, the mice were randomized according to tumor volume (six mice per group). Mice in the administration group were intraperitoneally injected with Chr (20 or 40 mg/kg) or PD-1 inhibitor (10 mg/kg, Bio X Cell) every other day for 12 days. The tumor volumes and body weights of mice were recorded every 2 days.

**Tissue processing and flow cytometry**

Bone marrow cell suspensions were obtained by flushing from mouse femurs and tibias. The spleen was squeezed to disperse through a 100 μm filter. The fresh tumor tissue removed from mice was digested with 1 mg/mL collagenase type I (Sigma-Aldrich, USA) and 50 units/mL DNase (Sigma-Aldrich, USA) for 3 h to obtain tumor single-cell suspension. Red blood cells (RBCs) were subsequently lysed using RBC lysis solution (Solarbio, China). After washing with 1% BSA (Solarbio, Beijing, China), the cells were blocked by non-specific staining with Fc block (BD Biosciences, USA). MDSCs were stained with fluorescence-conjugated antibodies to CD11b, Ly6G and Ly6C (eBioscience, USA). T-cells were stained for CD45, CD3, and CD8 (eBioscience, USA). Flow cytometric analysis was performed by using BD LSRFortessa and analyzed with FlowJo Software (Treestar).

**Mass spectrometry**

Samples were processed by EasyPep Mini MS Sample Prep Kit (Thermo scientific) following the manufacturer recommendation. MDSCs sorted from mouse bone marrow were lysed for quantification. After reduction and alkylation, the protein was digested with trypsin into peptides. The peptides were then purified and dried in a vacuum centrifuge. Finally, peptides were resuspended in 0.1% formic acid, and detected by Triple-quad Ion-trap and Orbitrap mass spectrometer (Thermo Scientific, USA). Pathway enrichment analyses were performed with Metascape (http://metascape.org)^[1]^.

**Cell cycle and apoptosis detection**

The MDSCs from the marrow of B16-F10 tumor-bearing mice were treated with Vehicle, Chr-low (10 μM), and Chr-high (20 μM) for 48h, respectively. For cell apoptosis analysis, the cells were stained according to the manufacturer’s instruction of Annexin V/PI apoptosis detection kit (KeyGen Biotech, China). Cell cycle analysis was performed using PI staining after drug treatment. Finally, cell apoptosis and cycle distribution were analyzed via flow cytometry (LSR Fortessa, USA).

**Cell proliferation assay**

MDSCs were stained with CFSE fluorescent dye (Sigma, USA) for 15 min. After the addition of the drug, the cells were incubated for 48 h, fluorescent signals were measured using flow cytometry (LSR Fortessa, USA).

**Arg-1 activity assay**

Follow the manufacturers instructions for the arginase activity assay kit (Abcam, UK). The drug-treated cells were collected, 10^6^ cells per group, washed with cold PBS, and resuspended in Assay Buffer. After centrifugation, the supernatant was transferred, mixed with the substrate, and incubated at 37°C for 20 minutes. Add the mixture containing OxiRed Probe. Measure the absorbance at 570nm.

**ROS assay**

The intracellular ROS generation was detected using a ROS assay kit (Beyotime, China) according to the manufacturers instructions. After the cells were treated with drugs for 48 hours, DCFH-DA diluted in serum-free medium was added, and the cells were mixed and incubated at 37°C for 20 minutes. ROS levels were detected by flow cytometry.

**Nitric oxide (NO) detection assay**

NO measurements were performed with the Griess reaction (Solarbio, China). NO levels were calculated by measuring absorbance at 550 nm.

**Quantitative real-time PCR**

The total RNA extracted from the cells was reverse transcribed into cDNA by using a PrimeScript RT reagent kit (Tiangen, China). Q-PCR with reverse transcription (RT–qPCR) of Arg-1, COX-2, iNOS, and GAPDH was performed using the Green RT–qPCR kit (Yeasen, China). The primers used for qPCR are listed in Table S1.

**Active Rho Pull-Down and Detection**

Follow the manufacturers instructions for active rho pull-down and detection kit (Thermo scientific, USA) to collect GTP-bound RhoA (RhoA-GTP) in cells. The active GTP-bound RhoA was detected using the RhoA antibody.

**Western blot**

Cell lysates of MDSCs were prepared with RIPA buffer 48 hours after drug treatment. Equal amounts of total protein were separated using 10% SDS–polyacrylamide gel and transferred onto the PVDF membrane. Membranes were blocked with 5% nonfat dry milk and incubated with primary antibodies against Arg-1 (1:1000, Proteintech, China), iNOS (1:1000, Proteintech, China), COX-2 (1:1000, Affinity, USA), RhoA (1:5000, Abcam, USA), AKT (1:1000, Affinity, USA), p-AKT (1:1000, Affinity, USA), and GAPDH (1:5000, Affinity, USA) at 4 °C overnight. After washes, membrane incubated with secondary antibodies (1:5000, Affinity, USA) for 2 h at room temperature. Detection was performed using an enhanced chemiluminescence kit (Vazyme, Nanjing, China).

**Histology and Immunohistochemistry**

The tumors were fixed with 10% paraformaldehyde for 48 h, embedded in paraffin, and cut into 4 μm sections for HE and IHC staining. HE staining was conducted according to routine protocols. For IHC, after using 3% H_2_O_2_ to block endogenous peroxidase activity, the deparaffinized sections were boiled in sodium citrate buffer for the antigen retrieval of IHC. The primary antibody (HIF-1α) was used in a dilution of 1:200 for 4 °C overnight. Finally, the envisioned system HRP-DAB kit (Beyotime Biotechnology) was used according to the manufacturer’s instructions.

**In Vivo Vascular Leakage and Perfusion Assay**

Tumor vascular leakage was analyzed following the intravenous injection of 100 μL of rhodamine-conjugated dextran (25 mg/mL, 70 kDa, Sigma-Aldrich) 30 min before the animal was sacrificed. For vascular perfusion studies, 100 μl of DyLight® 594-conjugated tomato lectin (1 mg/mL, Vector laboratory) was i.v. injected 30 min before the animal was sacrificed. Tumors were isolated and embedded with OCT; cryosections were cut to 10 μm thickness in a cryostat. Frozen tumor sections were fixed, blocked, and incubated with a CD31 antibody followed by a fluorescent secondary antibody. The specimen was observed with a confocal fluorescence microscope (Nicon, Japan).

**Statistical analysis**

Data were presented as mean ± SD and analyzed with GraphPad Prism software 8 (La Jolla, CA). The *p* values were assessed through unpaired two-tailed t test or one-way analysis of variance with Dunnett’s test. Differences in survival were analyzed using the Kaplan–Meier method with the log-rank test. Statistical significance was set at *P < 0.05 or **P < 0.01.

**References**

[1] ZHOU Y, ZHOU B, PACHE L, et al. Metascape provides a biologist-oriented resource for the analysis of systems-level datasets [J]. Nat Commun, 2019, 10(1): 1523.

Table S1. Primer sequences of q-PCR

| GAPDH-F | AACTTTGGCATTGTGGAAGG |
| --- | --- |
| GAPDH-R | ACACATTGGGGGTAGGAACA |
| Arg1-F | GCTGTCTTCCCAAGAGTTGGG |
| Arg1-R | ATGGAAGAGACCTTCAGCTAC |
| COX2-F | CCAGCACTTCACCCATCAGTT |
| COX2-R | ACCCAGGTCCTCGCTTATGA |
| iNOS -F | AACGGAGAACGTTGGATTTG |
| iNOS -R | CAGCACAAGGGGTTTTCTTC |
